# Atypical Presentation of Optic Neuritis in Multiple Sclerosis

**DOI:** 10.7759/cureus.76169

**Published:** 2024-12-21

**Authors:** Marwa Mukhtar, Mohib Naseer, Malik Mudasser Yasin, Faizan Luqman

**Affiliations:** 1 Ophthalmology, Medical Teaching Institution (MTI) Ayub Teaching Hospital, Abbottabad, PAK; 2 Ophthalmology, Medical Teaching Institution (MTI) Khyber Teaching Hospital, Peshawar, PAK; 3 Ophthalmology, University Hospital Limerick, Limerick, IRL; 4 Ophthalmology, Cork University Hospital, Cork, IRL; 5 Ophthalmology, Khyber Medical College, Peshawar, PAK

**Keywords:** atypical optic neuritis, autoimmune disease, mri, multiple sclerosis, typical optic neuritis

## Abstract

Optic neuritis (ON) is the inflammation of the optic nerve. ‘Typical’ ON is commonly associated with multiple sclerosis (MS) and its classic triad includes sudden loss of vision, pain with eye movement and dyschromatopsia. It usually has good visual outcome irrespective of treatment. Presentation other than this is termed as ‘atypical’ ON and causes include autoimmune diseases, infections and optic neuropathy related to systemic disorders, etc. These cases of ON need specific treatment depending on the cause.

Therefore, it is vital to differentiate ‘typical’ from ‘atypical’ ON. Here, we present a case of ON associated with MS, which presented with atypical symptoms i.e. painless loss of vision. Moreover, this patient had a poor final visual outcome, in contrast to the generally good visual prognosis of ON associated with MS.

## Introduction

Optic neuritis (ON) refers to the inflammation of the optic nerve [1]. The incidence of ON in central Europe is 5 cases per 100,000 persons per year. The majority (more than 70%) of patients are women and the average age at onset is 36 years [2]. Causes of optic neuritis include demyelination, autoimmune diseases, infections, idiopathic and optic neuropathy related to systemic diseases, etc.

The most common cause of optic neuritis is demyelination related to multiple sclerosis (MS), which is often termed as ‘typical’ ON [1]. ON is the initial presentation in about 20% of cases of MS [3] and may occur in 50% of cases of MS during the course of the disease [4]. The usual presentation of ‘typical’ ON is acute, painful, unilateral vision loss with a good visual prognosis even without treatment, though steroids can be administered to expedite visual recovery.

In contrast to this, ‘atypical’ optic neuritis needs specific treatment depending upon the cause and can have severe visual consequences if not treated timely. Causes of atypical optic neuritis include neuromyelitis optica (NMO), autoimmune optic neuropathy, optic neuropathy related to systemic disease and idiopathic causes [1].

## Case presentation

A 48-year-old female patient presented to eye casualty with sudden painless loss of vision in the left eye. She had no known past ocular and medical history. On examination, her best corrected visual acuity (BCVA) was 6/6 in the right eye and hand movement (HM) in the left eye. She had left relative afferent pupillary defect (RAPD). Her extraocular movements were intact. Color vision was normal in the right eye and severely affected in the left eye (could not read any plates). Anterior and posterior segment examination was normal with no optic disc swelling. The rest of the neurological examination was normal, with no signs of neurological deficit.

**Figure 1:**
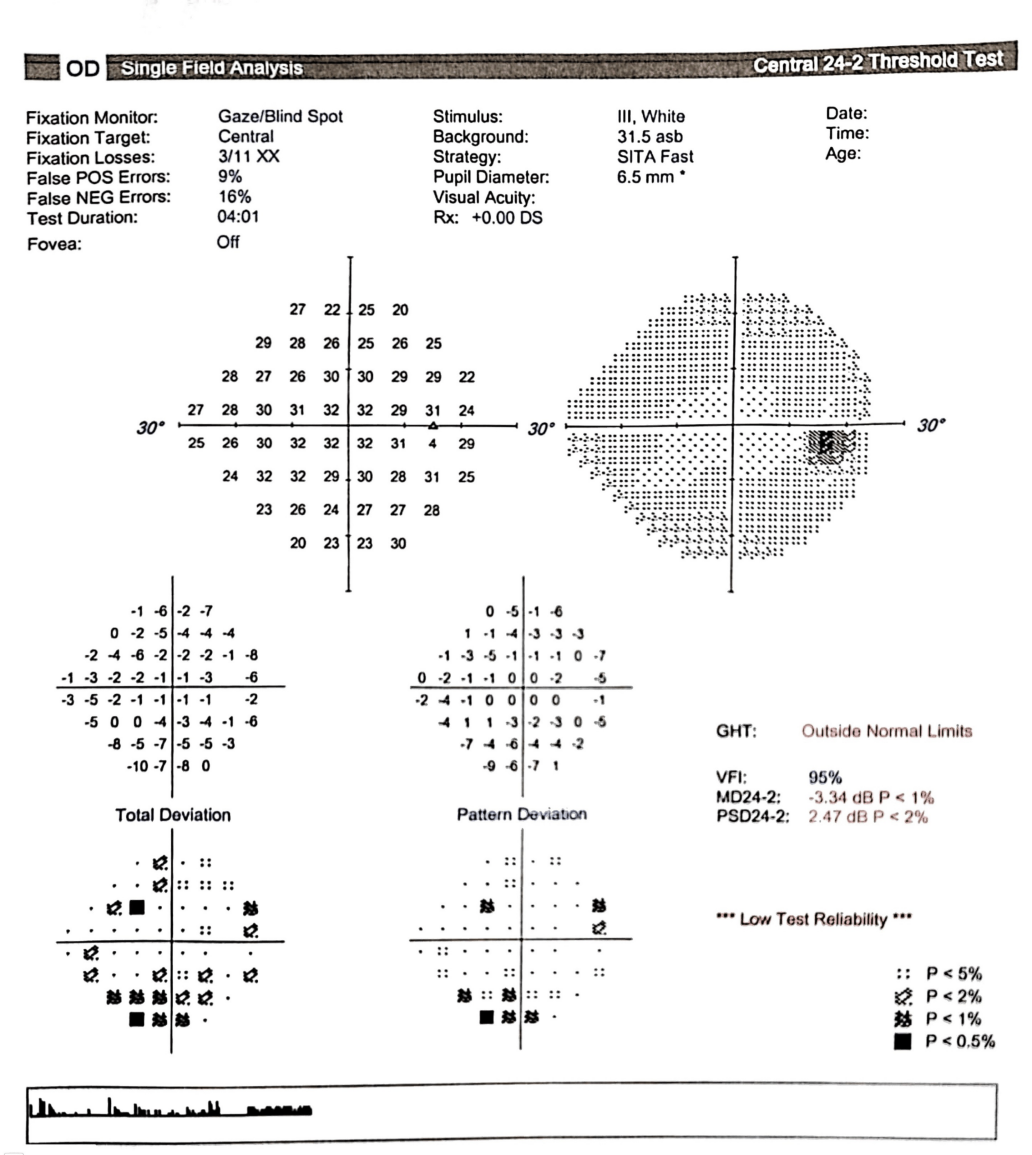
Humphrey visual field (24-2) of the right eye showing no gross visual field defect

**Figure 2:**
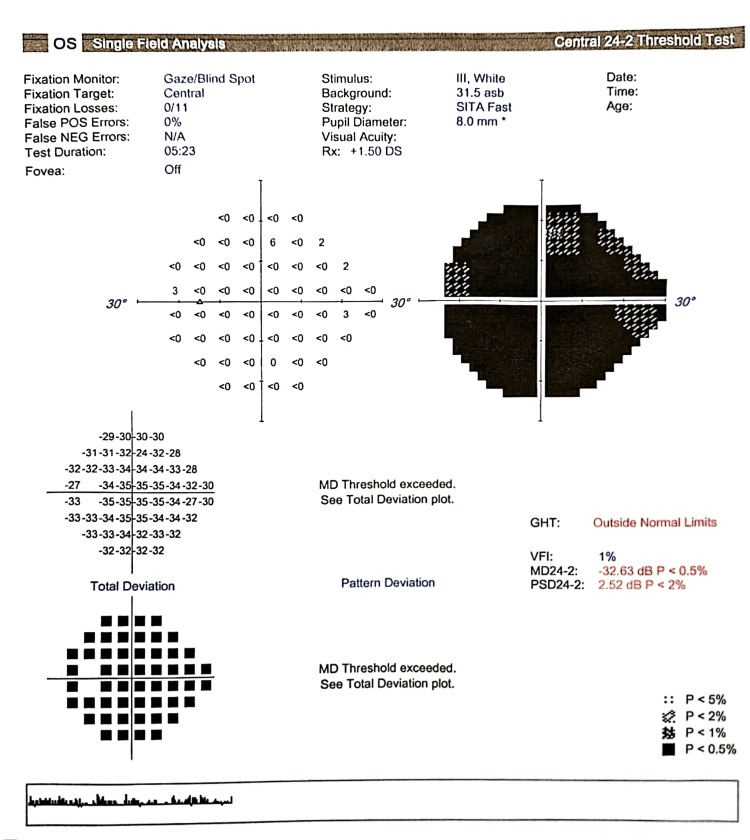
Humphrey visual field (24-2) of the left eye

Her visual field test was normal in the right eye and was not possible to assess in the left eye (Figures 1, 2).

She was referred to the acute medical unit for further evaluation by the neurological team and investigations to rule out the causes of retrobulbar optic neuritis and ischemic optic neuropathy.

MRI (brain and orbit) with contrast T2/FLAIR, showed callososeptal hyperintense plaque in the left frontal lobe and juxtacortical plaque in the right frontal lobe. The callososeptal and juxtacortical lesions were in a typical distribution of demyelination. The left optic nerve (retrobulbar) appeared larger than the right, showing asymmetry of both optic nerves. These findings met the criteria of dissemination in space for MS. Her lumbar puncture showed oligoclonal bands in cerebrospinal fluid (CSF). All other autoimmune screening tests were normal including anti-MOG antibodies and anti-aquaporin-4 antibodies.

Moreover, other investigations to rule out co-existent ischemic events were normal including echocardiography and carotid Doppler ultrasound scan.

She received intravenous steroids followed by oral steroids (intravenous methylprednisolone 1 gram/day for three days, followed by oral prednisolone 1 mg/kg/day for 11 days with a quick taper over five days) and was reviewed in the eye clinic after eight weeks. On a follow-up visit, visual acuity improved minimally in the left eye, from HM to 1/60 only. She still had left RAPD with severely affected color vision. On fundoscopy, the left optic nerve appeared pale and atrophic.

## Discussion

Optic neuritis is defined as inflammation of the optic nerve which can be classified as ‘typical’ and ‘atypical’ ON. Most common cause of ‘typical’ ON is demyelination, associated with multiple sclerosis [1]. In 50% of cases with MS, optic neuritis may present during the course of the disease and ON is a presenting feature of MS in 20% of patients [4]. The classic triad includes visual loss, periocular pain and dyschromatopsia and has a good visual prognosis even without treatment [5].

MS is a debilitating disease and the risk of MS after optic neuritis may be predicted [6]. McDonald criteria is commonly used for the diagnosis of MS, which includes clinical features and specific magnetic resonance imaging (MRI) findings. Diagnostic criteria include dissemination of demyelinating lesions in space (DIS) and time (DIT), in the central nervous system [7].

Earlier and aggressive treatment in optic neuritis can reduce the risk of later development of clinically definite MS [6]. According to the Optic Neuritis Treatment Trial (ONTT), intravenous methylprednisolone speeds up visual recovery and reduces the frequency and severity of MS, but has no effect on final visual outcome [8]. Earlier therapy with corticosteroids in ON reduces the risk of permanent neuronal damage and therefore progressive disability in MS [6]. Therefore, an accurate diagnosis of ON is critical and risk assessment should be done regarding the possibility of conversion to MS [4]. It is crucial to diagnose the cause of ON, as ‘atypical’ cases need different management.

The patient presented here with ‘atypical’ symptoms i.e. painless loss of vision but investigations met the criteria of MS (dissemination in space-DIS). As discussed above, ON associated with MS usually has good visual outcomes irrespective of intravenous steroid treatment, but she had minimal visual recovery (HM to 1/60) at eight-week follow-up visit.

## Conclusions

This is a case of ‘atypical’ presentation of optic neuritis associated with multiple sclerosis. Therefore, every case of ON, including atypical symptoms, should be thoroughly investigated to rule out other possible causes of ON and its management accordingly. Furthermore, this case study shows that ‘atypical’ cases of ON associated with MS, can have an unfavorable visual outcome, in contrast to the generally good visual prognosis of ON associated with MS and patients should be counselled regarding possible adverse visual outcomes.
